# Unlocking the Therapeutic Potential of Integrin‐Linked Kinase Inhibitors in Bioengineered 3D Breast Tumor Stroma Models

**DOI:** 10.1096/fj.202503695R

**Published:** 2026-01-15

**Authors:** Salma T. Rafik, Anuja Upadhyay, Alexander J. MacRobert, Umber Cheema

**Affiliations:** ^1^ UCL Centre for 3D Models of Health and Disease, UCL Division of Surgery and Interventional Science, Faculty of Medical Sciences Charles Bell House London UK; ^2^ Department of Clinical Pharmacology, Faculty of Medicine Alexandria University Alexandria Egypt

**Keywords:** 3D tumoroids, breast cancer, Integrin‐linked kinase, tissue engineering, tumor microenvironment, tumor‐stroma

## Abstract

The tumor microenvironment (TME) plays a pivotal role in breast cancer progression and metastasis, and the efficacy of targeted therapies is influenced by the heterogeneous nature of the TME. Interactions between breast cancer cells and their surrounding stromal cells modulate proliferation, invasion, and survival pathways, often via integrin‐mediated mechanotransduction and growth factor signaling. Integrin‐linked kinase (ILK) is a serine/threonine protein kinase that has been widely established as a critical driver of breast cancer progression, metastasis, and therapeutic resistance. Its expression is frequently upregulated in breast cancer tumors and correlates with poor prognosis. Given that ILK activity is highly dependent on cell‐matrix interactions that are only recapitulated in 3D culture, we investigated the effect of an ILK inhibitor in 3D bioengineered compartmentalized breast tumoroid models to better mimic in vivo conditions. Two tumor cell masses (MDA‐MB‐231 or MCF‐7) were cultured within a primary breast tissue stromal compartment representative of breast tissue or a metastatic representative of lung tissue. In highly invasive and highly hypoxic MDA‐MB‐231 3D tumoroid models, ILKI treatment was 2.2 fold more effective in 3D models representative of breast tissue (*p*‐value < 0.0001) compared to those with the metastatic lung compartment (*p*‐value = 0.03). However, ILKI treatment was slightly more effective (1.4 fold) in the less invasive and less hypoxic MCF‐7 3D tumoroid models with the metastatic lung compartment compared to those with the primary breast compartment. Non‐invasive imaging of oxygen gradients in the 3D models shows alleviation of hypoxia following treatment and correlation with enhanced treatment efficacy. These results emphasize the necessity of modeling both the tumor and the stroma since this interaction can directly influence drug efficacy. Moreover, ILK inhibitor treatment holds promise for breast cancer therapy particularly in chemotherapeutic resistant cases.

## Graphical abstract

3D cancer models provide a more reliable representation of drug responses compared to 2D cell culture owing to their 3D architecture and facilitation of cell‐extracellular matrix interactions. In this study, we engineered dense compartmentalized 3D tumoroids composed of breast tumor mass and a surrounding stromal compartment replicating either breast tissue or metastatic lung site. The developed 3D tumoroids enabled investigating the efficacy of an integrin linked kinase inhibitor and comparing its effect on the tumor mass and the stromal compartment. Real‐time oxygen gradient monitoring in the 3D tumoroids was also utilized as a method for assessing drug efficacy. These 3D models are suitable for therapeutic screening in breast cancer, particularly therapies targeting the tumor microenvironment. Our research highlights the potential of Integrin‐linked kinase inhibitors as candidates for the treatment of breast cancer.

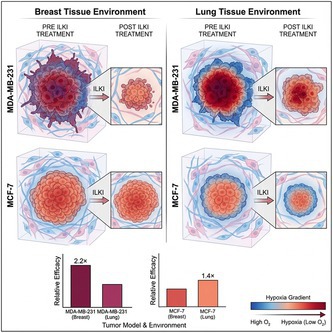

**Abbreviations**
BC: breast cancer
CAFs: cancer‐associated fibroblasts
CL: Claudin low
COX‐2: cyclooxygenase‐2
EC: endothelial cells
ECM: extracellular matrix
EGF: epidermal growth factor
EMT: epithelial‐to‐mesenchymal transition
ER: estrogen receptors
FGF: fibroblast growth factor
GSK3β: glycogen synthase kinase‐3 beta
HER2: human epidermal growth factor receptor 2
HGF: hepatocyte growth factor
HNLF: human neonatal lung fibroblast
HUVEC: human umbilical vein endothelial cell
IGF‐1: insulin‐like growth factor 1
IL‐6: interleukin‐6
ILKI: Integrin‐linked kinase inhibitor
LVER: linear viscoelasticity region
MAP‐kinase: mitogen‐activated protein kinase
MMP: matrix metalloproteinase
MRTF: myocardin‐related transcription factor
MSCs: mesenchymal stromal cells
NF‐κB: nuclear factor kappa‐light‐chain‐enhancer of activated B cells
PDGF: platelet‐derived growth factor
PGE2: prostaglandin E2
PINCH: particularly interesting new cysteine‐histidine–rich protein
PKB: protein kinase B
PR: progesterone receptors
SDF‐1: stromal cell‐derived factor 1
TGF‐β: transforming growth factor beta
TM: tumor mass
TME: Tumor microenvironment
TNBC: triple‐negative breast cancer
VEGF: vascular endothelial growth factor
YAP/TAZ: yes‐associated protein/transcriptional coactivator with PDZ‐binding motif

## 1 Background

Breast cancer continues to be the most common cancer and a leading cause of cancer mortality in women worldwide. It is a diverse disease with various clinical and molecular characteristics [1]. Recent advancements in molecular profiling have classified breast cancer into a minimum of six distinct subtypes, which include luminal A, luminal B, HER2‐enriched, normal‐like, Claudin‐Low (CL), and basal‐like/triple‐negative breast cancer (TNBC) [2]. Each subtype of breast cancer is associated with unique clinical outcomes and varying responses to treatment [3]. Luminal A tumors (estrogen receptors (ER)+, progesterone receptors (PR)+) are typically characterized by the best prognosis [4], whereas TNBC, which lacks the expression of ER, PR, and HER2, frequently correlates with the poorest prognosis [5]. CL tumors represent a highly aggressive and metastatic type of TNBC owing to their pronounced transcriptional repression of genes critical for epithelial integrity, specifically integral tight junction components, including claudin‐3, claudin‐4, and claudin‐7, and occludin, alongside the critical adherens junction protein, E‐cadherin [6].

The complexity of breast tumors is closely associated with the tumor microenvironment (TME), rather than solely reliant on the intrinsic characteristics of the tumor cells [7]. TME is a dynamic ecosystem that evolves in response to disease progression. It consists of various cellular elements, including tumor cells and an array of non‐tumorous cells such as mesenchymal stromal cells (MSCs), immune cells, fibroblasts, endothelial cells, adipocytes, in addition to non‐cellular physical and chemical factors [8]. These factors encompass gradients of oxygen, nutrients, and pH, as well as signaling molecules and components of the extracellular matrix (ECM). The components of the TME play a crucial role in tumor initiation, the formation of pre‐metastatic niches, the metastatic process, and the tumor response to various challenges, including nutrient and oxygen deprivation as well as therapeutic agents [9]. TME components are involved in the development of drug resistance through complex molecular interactions [10]. The TME is actively shaped by various stromal cell populations. Specifically, immune cells drive tumor progression by fostering immunosuppression and facilitating the metastatic cascade [11]. Analogously, cancer‐associated fibroblasts (CAFs) augment cancer cell longevity and malignant potential via the release of paracrine growth factors and cytokines, concurrently constructing a “protective niche” that shields against therapeutic agents [11]. Additionally, adipocytes and MSCs can further promote matrix remodeling and tumor survival through their secretome [11]. ECM is increasingly acknowledged as a vital regulator of breast tumorigenesis, metastatic behavior, and the development of chemoresistance [12]. Cancer cell adhesion to ECM components like collagen, laminin and fibronectin, or growth within a stiff matrix, promotes chemotherapy resistance. ECM‐driven resistance stems mainly from integrin signaling activation, leading to increased pro‐survival and anti‐apoptotic proteins, altered drug efflux, and phenotypic changes (e.g., epithelial‐to‐mesenchymal transition [EMT] or cancer stemness), a phenomenon called cell adhesion‐mediated drug resistance. Integrin‐linked kinase (ILK) is an important mediator of signal transmission from the ECM. It interacts with β1‐integrin, facilitating the transfer of extracellular signals from the ECM, thereby regulating cellular functions. A study reported that β1‐integrins are key mediators of Collagen 1‐related doxorubicin resistance in MCF‐7 cells and MDA‐MB‐231 cells [13]. Consequently, TME has emerged as a significant prognostic and predictive biomarker for breast cancer. Evidence suggests that therapies targeting TME interactions may revolutionize cancer treatment. Many preclinical and clinical studies targeting TME components demonstrate significant reduction in tumor growth, metastasis, and chemoresistance [14, 15].

Although in vitro two‐dimensional (2D) cell culture models have been commonly utilized in breast cancer studies, they display limited biomimicry and do not properly recapitulate breast cancer complexity. Three‐dimensional (3D) cell culture models better mimic the physiological 3D tissue architecture of the TME. Herein, 3D breast tumor models engineered using dense collagen I gels were configured with spatial compartmentalization of a tumor mass (TM) within a stromal compartment simulating the tumor and TME. The stroma was engineered to either mimic the primary tissue stroma (adipose derived stromal cells with an endothelial network), or the metastatic lung stroma (human lung fibroblasts and an endothelial network). Compartmentalized 3D culture systems are highly advantageous as they permit the precise tuning of the ECM's density and composition, thereby enabling the study of physiologically relevant cell–cell and cell‐matrix interactions that mimic either primary tissue or metastatic stroma. Furthermore, the stiffness and density of the collagen within these models not only dictate cellular growth and morphology but also naturally create a hypoxic core—a phenomenon frequently observed in vivo resulting from restricted oxygen transport through the denser scaffold [16].

ILK's role in tumorigenesis is fundamentally regulated by the physical and biochemical cues of the extracellular matrix such as ECM stiffness, integrin signaling, and oxygen deprivation. As these cues are largely absent in traditional 2D culture and because ILK is recognized as a critical driver of breast cancer cell proliferation, survival, and metastasis, we utilized a complex 3D hydrogel culture system to specifically investigate the therapeutic effect of an ILK inhibitor (ILKI) in a physiologically matrix relevant 3D breast tumor model at either the primary tumor site or a common metastatic site, the lung.

## 2 Methods

### 2.1 Cell Culture

Human breast cancer cell lines MCF‐7 and MDA‐MB231 were sourced from the European Collection of Authenticated Cell Cultures (ECACC). Both cell lines were routinely propagated in Dulbecco's Modified Eagle's Medium/Nutrient Mixture F12 Ham (DMEM/F12) growth medium (Sigma Aldrich, Dorset, UK) supplemented with 10% FBS (Gibco through Thermo Fisher Scientific, Loughborough, UK), and 1% penicillin (5000 units/mL)/streptomycin (5000 μg/mL) (Gibco through Thermo Fisher Scientific, Loughborough, UK). Human normal lung fibroblasts (HNLF) were obtained from Lonza, Bioscience. Adipose tissue‐derived mesenchymal stem cells (MSCs) were acquired from the American Type Culture Collection located in Virginia, United States (ATCC). These cells were cultured in Dulbecco's Modified Eagle Medium (DMEM) containing 1 g/L D‐glucose (Gibco, Thermo Fisher Scientific, Loughborough, UK), supplemented with 10% FBS, and 1% penicillin (5000 units/mL)/streptomycin (5000 μg/mL). Human umbilical vein endothelial cells (HUVECs) were procured from Promocell in Heidelberg, Germany, and were maintained in endothelial growth media (Promocell) supplemented with 10% FBS and 1% penicillin/streptomycin. All cell propagation and experimentation were conducted under rigorously controlled environmental parameters of 5% carbon dioxide (CO_2_) and 95% atmospheric air at 37°C to ensure optimal cellular physiology.

### 2.2 Fabrication of In Vitro 3D Breast Cancer Model (Tumoroids)

Fabrication of 3D tumoroids was achieved using the RAFT protocol (Lonza, Basel, Switzerland), which generates collagen‐based constructs with physiologically relevant densities using the plastic compression method [17]. This entire procedure was done under aseptic conditions and kept on ice to prevent premature collagen polymerization.

#### 2.2.1 Tumor Mass

Tumor cell microtissues were manufactured by preparing a cellular suspension containing 2 × 10^4^ cells/gel of either MCF‐7 or MDA‐MB‐231 combined with the following components: 10 × MEM (Gibco through Thermofisher Scientific, Loughborough, UK), type I collagen 2 mg/mL (First Link, Birmingham, UK), and 10 μg/mL laminin. pH neutralization was achieved using a solution composed of 17% 10 M NaOH (Sigma‐Aldrich, Dorset, UK) and 1 M HEPES buffer (Gibco through Thermofisher Scientific, Loughborough, UK). Subsequently, 240 μL aliquots of the cell‐collagen mixture were pipetted into 96‐well plates (Corning through Thermofisher Scientific, Loughborough, UK) and allowed to polymerize via incubation at 37°C for 15 min. The resulting constructs underwent plastic compression using specialized absorbers for 15 min. Finally, the compressed gels were overlaid with 100 μL of cell culture media.

#### 2.2.2 Two‐Compartment Tumoroids

**FIGURE 1:**
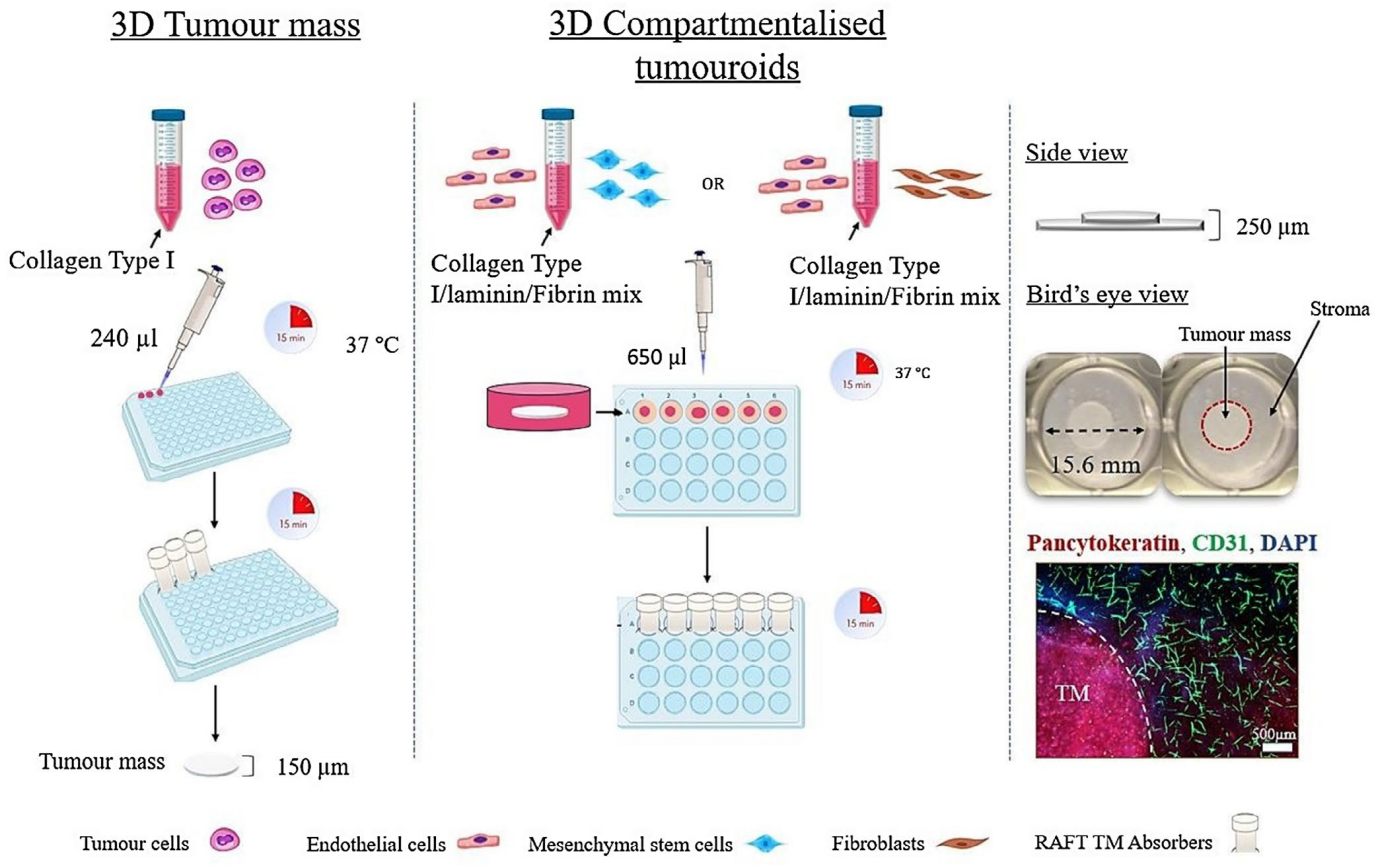
Method of fabrication of 3D tumoroids by plastic compression of collagen type I hydrogels using RAFT absorbers. The 3D compartmentalized tumoroids consist of a central mass of breast cancer cells surrounded by a stromal compartment containing endothelial cells with either mesenchymal stromal cells (Primary stroma) or human lung fibroblasts (Metastatic stroma). TM, tumor mass.

The development of compartmentalized tumoroids involved embedding the TM within a defined stromal compartment. The stromal compartment gel solution was engineered by mixing specific ECM constituents and the relevant stromal cell populations in a collagen‐based solution similar to that formulated for the TM, as previously mentioned. In a 24‐well plate (Corning through Thermo Fisher Scientific, Loughborough, UK), an initial 650 μL stromal layer was polymerized at 37°C for 15 min followed by placement of the prefabricated TMs on top of the set gels and addition of a second 650 μL of the stromal layer, then incubated for a further 15 min at 37°C. Afterward, the fluid was removed by using the 24‐well RAFT absorbers at room temperature for 15 min to increase the hydrogel density. All the produced constructs were maintained in 1 mL of cell culture media. 3D compartmentalized tumoroids of MCF‐7 and MDA‐MB‐231 were engineered to include two distinct stromal compartments that represent primary breast cancer and metastatic breast cancer in the lungs (Figure 1).

- *Primary breast stromal compartment*: the cellular component of the stromal niche was seeded with a co‐culture population consisting of 1 × 10^4^ MSCs and 1 × 10^5^ HUVECs. The ECM base was established through the addition to 2 mg/mL fibrin and 20 μg/mL laminin (Sigma‐Aldrich, Dorset, UK).
- *Metastatic breast stromal compartment*: the cellular component of the stromal compartment comprised 1 × 10^4^ HNLF and 1 × 10^5^ HUVECs in addition to the matrix components of 2 mg/mL fibrin and 20 μg/mL laminin.

Over the course of 21 days, compartmentalized tumoroids are cultured at 37°C under 5% carbon dioxide (CO_2_) and 95% atmospheric air with 50% of the culture media changed every 48 h.

##### 2.2.2.1 Measurement of Cancer Invasion and Vascular Networks Formation

On Day 21, the samples were fixed using 4% formalin, followed by immunostaining with an anti‐pancytokeratin (Genetex Inc., US), anti‐platelet cell adhesion molecule (CD31) (Abcam, UK), and DAPI. Following this, fluorescence imaging was conducted using the Zeiss AxioObserver with ApoTome.2 instrument and software (Zeiss, Oberkochen, Germany). Cancer invasion was assessed by measuring the surface area of invasion into the surrounding stroma in images taken at four positions, 12, 3, 6, and 9 o'clock on the same focal plane for each sample. The images were subsequently uploaded to Fiji (NIH open‐source software). The total surface area of invasion for each sample was calculated. The complexity and morphology of the endothelial structures present within the stromal compartment were assessed through comprehensive image analysis to determine several key morphological parameters including the counts of loops, junctions, and branch points alongside the quantification of the total branch lengths. All resultant data were subjected to statistical analysis using GraphPad Prism 9 software.

##### 2.2.2.2 Evaluation of Oxygen Consumption in Tumoroids

A luminescence‐based optical oxygen mapping technique provided by the VisiSens TD MIC System was utilized to assess spatio‐temporal distribution of oxygen within the tumoroids. The underlying principle of the technique entails the collection of luminescent signals emitted by an optical sensor foil, with the signals subsequently captured via 2D imaging. The 3D tumoroids were maintained in 1 mL of cell culture media in a 24‐Well imaging sensor plate (model ISP24‐RPSu4, PreSens Precision Sensing GmbH, Regensburg, Germany) equipped with adhered oxygen sensor foils. The 24‐well plate was incubated at 37°C throughout the experiment. Signal acquisition was established as a 24‐h time lapse series, with data collected every hour. The system was calibrated to define the 0% and 100% oxygen reference points through utilizing a 1% (wt/V) sodium sulfite solution to represent 0% oxygen and distilled water to serve as 100% oxygen. Data processing was conducted using the VisiSens ScientifiCal Software Version VS 1.0.1.5 Plugins, wherein the TM was designated as the region of interest for analysis. The collected data was graphically represented as the analyte percentage versus time. Once the oxygen consumption reached a steady‐state level, the average oxygen percentage within the ROI was calculated and subsequently analyzed for statistical significance using GraphPad Prism 9 software.

### 2.3 Experiments in 2D

The cytotoxic effects of the ILK inhibitor “OSU‐T315” (MedChemExpress through Insight Biotechnology Limited, UK) were assessed in 2D cultured MCF‐7, MDA‐MB‐231, HUVECs, MSCs, and HNLF in a 96‐well plate at a density of 4 × 10^3^ cells per well. Subsequently, the cells were subjected to 48 h of incubation with increasing concentrations of OSU‐T315, ranging from 0.2 to 3 μM. Cell viability was measured using the CellTiter‐Glo 3D Viability assay following the protocol described below.

### 2.4 Experiments in 3D

Drug intervention was carried out on Day 18 post‐seeding of the compartmentalized tumoroids. The compartmentalized tumoroids, featuring two distinct stromal compartments, were treated with 10 μM of OSU‐T315 for 48 h. Following the treatment period, the drug was thoroughly removed by performing two sequential washes with PBS, and fresh culture medium was subsequently replenished. The samples were incubated for an additional 24 h prior to conducting the endpoint involved testing different concentrations on samples of just TM.

#### 2.4.1 Cellular Viability Assessment

Cellular viability for both the 2D monolayer cultures and the 3D constructs was quantitatively determined by measuring intracellular ATP levels utilizing the CellTiter‐Glo 3D Viability Assay (Promega, Southampton, UK). Following the predetermined drug exposure period, the CellTiter‐Glo reagent was combined with the cell culture medium in a 1:1 ratio. The mixture was then subjected to vigorous agitation for 5 min on an orbital shaker to ensure complete lysis with subsequent incubation at room temperature for 25 min to stabilize the luminescence signal. Afterward, 100 μL of the reaction mixture was transferred in triplicate to an opaque black 96‐well plate (ThermoFisher Scientific, UK). Luminescence was measured using the Tecan Infinite Lumi plate reader (Männedorf, Switzerland).

#### 2.4.2 Fluorescence Microscopy (Live/Dead Dyes)

Fluorescence‐based cell viability analysis was performed on 3D compartmentalized tumoroids using the Live‐Dead Viability Kit from Molecular Probes (Thermo Fisher Scientific, United Kingdom). Following a PBS wash, the samples were incubated with the Live‐Dead mix solution, comprising 4 μM Calcein‐AM and 2 μM Ethidium Homodimer‐1, for 40 min at room temperature. Images were then acquired utilizing the Zeiss AxioObserver equipped with the ApoTome.2 instrument and software (Zeiss, Oberkochen, Germany), employing a filter for green (%Ex/Em 495/515 nm) and a red (%Ex/Em 495/635 nm) fluorescence detection.

#### 2.4.3 Analysis of Oxygen Concentration Gradients

A comparison of oxygen gradient levels in MCF‐7 or MDA‐MB‐231 3D compartmentalized tumoroids with different stromal compositions was carried out using real‐time oxygen monitoring using the VisiSens TD MIC System, as described before.

#### 2.4.4 Mechanical Testing of 3D Tumoroids

All mechanical measurements were performed using the Kinexus Pro+ rheometer (Netzsch, Germany), which performs stress‐controlled, shear rate‐ and strain‐controlled oscillatory testing. All tests used the roughened immersion well geometry and a 20 mm roughened steel plate top geometry.

The rheometer has a torque range of 1.0 nNm to 225 mNm and all samples were tested at a 0.2 mm gap to ensure a no‐slip boundary between the samples and geometry. Main measures for mechanical characterization were the shear elastic modulus (G'), linear viscoelasticity region (LVER), phase angle (δ), and the shape of the amplitude sweep curve.

Strain/amplitude sweep: An oscillatory strain sweep was conducted, where the shear stress increases from 0.1% to 100.0% and the amplitude refers to the maximum of the oscillatory motion. This testing was performed at 37°C with a constant frequency of 1.0 Hz. Amplitude sweeps determine the linear viscoelasticity region of a material's response to shear stress. 5 samples per decade were measured for each test and normal force is kept under 1 N.

### 2.5 Statistical Analysis

Data analysis was conducted using GraphPad Prism 9 software (GraphPad, San Diego, CA). Data normality was evaluated first through the Shapiro–Wilk test (*n* ≥ 3), followed by the application of the relevant statistical test for significance according to the parameters of the data set. The specific tests applied for each graph are detailed in the figure legends, and *p* values of < 0.05 were considered statistically significant.

## 3 Results

### 3.1 Presence of a Metastatic Stromal Compartment Significantly Enhances Cancer Invasion, Severely Impacts Vascular Networks Formation, and Limits Hypoxia Development in Breast Tumoroids

Emerging evidence has highlighted the significant role of tumor‐stroma crosstalk in promoting tumor growth. The first set of experiments was conducted to investigate the impact of stromal composition on breast tumor invasion, vascularization, and oxygen levels. A comparison between engineered compartmentalized tumoroids with three different types of stroma: (1) acellular, (2) MSCs and ECs, and (3) HNLF and ECs. MSCs in the stroma supported the development of extensive vascular networks, while no networks formed in the stroma seeded with HNLF cells (Figure 2.1). Engineered 3D compartmentalized tumoroids of MCF‐7 containing HNLF in the stromal compartment showed a significant increase in cancer invasion compared to those containing MSCs in the stroma (*p*‐value = 0.036) and acellular stroma (*p*‐value = 0.034). The total invasion area measured was 22.3 mm^2^ ± 2.9 for the acellular stromal tumoroids, which increased to 85.5 mm^2^ ± 5.2 in the MSCs containing tumoroids and 3592 mm^2^ ± 1290 in the HNLFcontaining tumoroids as shown in Figure 2.2. For 3D compartmentalized tumoroids of MDA‐MB‐231, the presence of HNLF in the stromal compartment resulted in a remarkable increase in cancer invasion compared to those containing MSCs in the stroma and acellular stroma (*p*‐value < 0.0001). A measured total invasion area of 7591 mm^2^ ± 112 in the tumoroids containing HNLF, 87 mm^2^ ± 22.8 in the tumoroids seeded with MSCs, and 125.5 mm^2^ ± 9.5 was found (Figure 2.2). Interestingly, the presence of vascularized stroma in MCF‐7 compartmentalized tumoroids containing MSCs led to a notable reduction in oxygen levels, dropping from 100% oxygen air saturation in MCF‐7 compartmentalized tumoroids with acellular stroma to around 25% in MCF‐7 compartmentalized tumoroids. On the other hand, MCF‐7 compartmentalized tumoroids containing HNLF showed less hypoxia, where the oxygen level reduced to 66.2% (Figure 2.3). This suggests that the metabolic activity and oxygen consumption in MCF‐7 3D compartmentalized tumoroids featuring primary stroma are elevated compared to those with metastatic stroma, potentially due to disparities in the stromal cells. MDA‐MB‐231 compartmentalized tumoroids with MSCs containing stroma showed slightly reduced oxygen levels compared to those containing HNLF.

**FIGURE 2:**
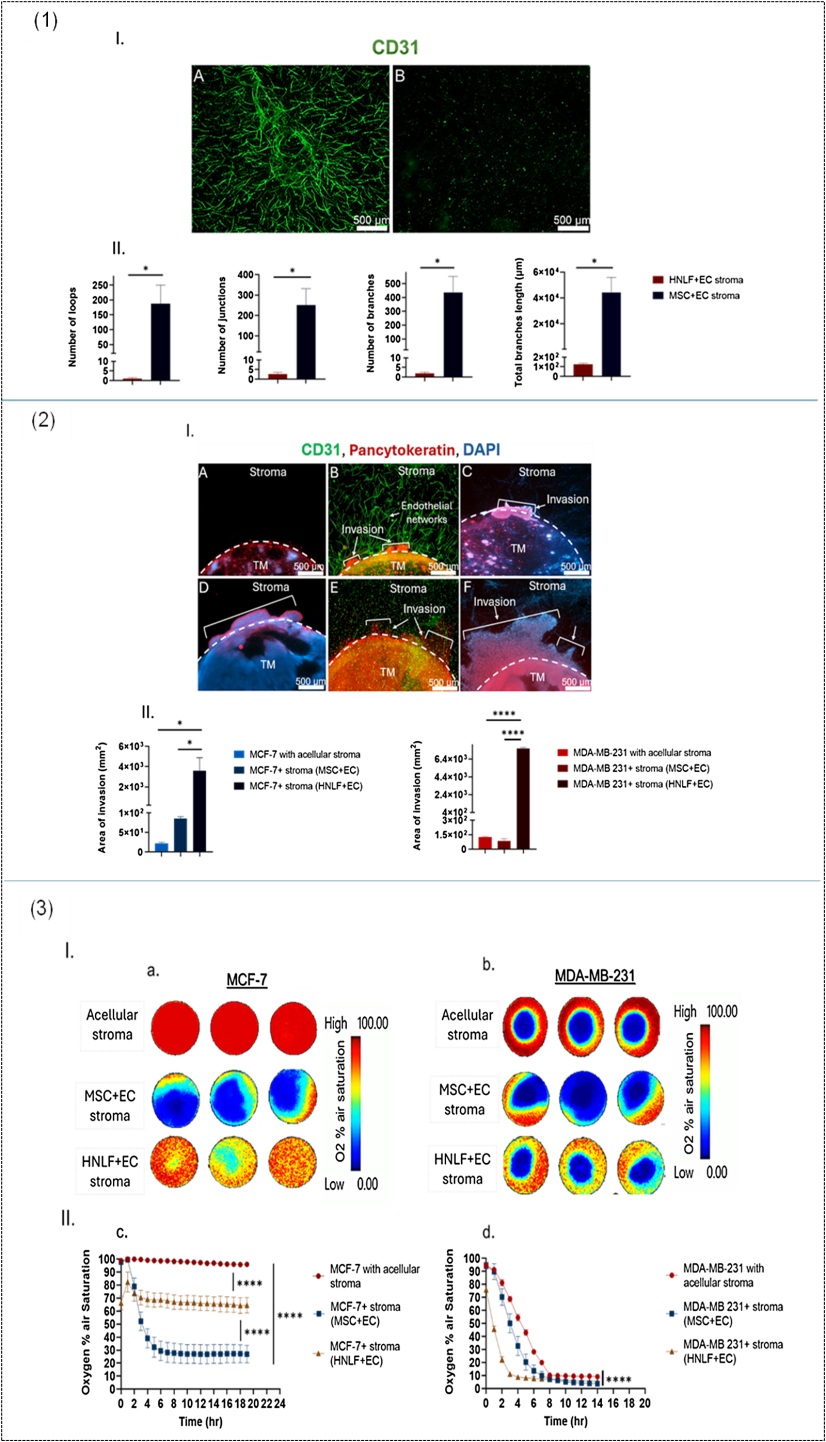
Impact of stromal composition on vascularization, cancer invasion and oxygenation in 3D compartmentalized tumoroids of MCF‐7 and MDA‐MB‐231. (1.I) A and B represent fluorescence images of the stromal compartment where A is composed of MSCs cells and ECs, and B is composed of HNLF and ECs. (1.II) shows a graph of quantified parameters of vascular networks complexity. (2.I) Upper panel shows fluorescence images of MCF‐7 3D compartmentalized tumoroids with (A) acellular stroma, (B) stroma containing MSCs and ECs or (C) stroma containing HNLF and ECs while the lower panel shows fluorescence images of MDA‐MB‐231 3D compartmentalized tumoroids with (D) acellular stroma, (E) stroma containing MSCs and ECs or (F) stroma containing HNLF and ECs respectively. (2.II) show graphs of the measured area of invasion in both MCF‐7 and MDA‐MB‐231 3D compartmentalized tumoroids. (3.I) shows pseudocolor images that capture the real‐time monitoring of oxygen gradient levels in (a) MCF‐7 3D compartmentalized tumoroids and (b) MDA‐MB‐231 3D compartmentalized tumoroids. (3.II) shows a graph of the recorded percentage of oxygen air saturation in (c) MCF‐7 3D compartmentalized tumoroids and (d) MDA‐MB‐231 3D compartmentalized tumoroids. *n* = 3, Unpaired *t*‐test. Data is shown in graphs as the mean and standard error of the mean. *p*‐values: *< 0.05, ****< 0.0001. TM, tumor mass.

### 3.2 Stiffness Measurements in 3D Breast Tumor Models

**FIGURE 3:**
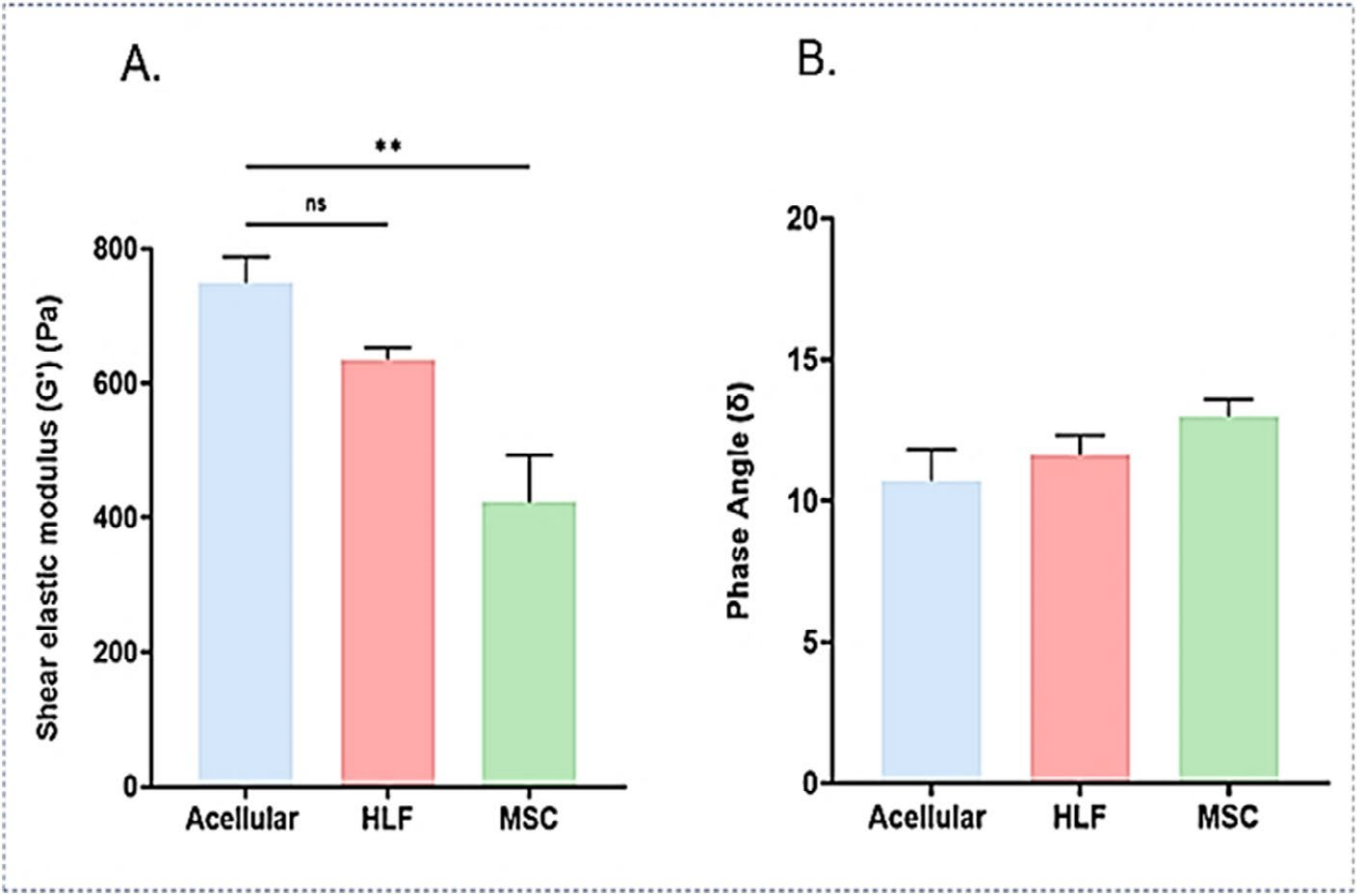
Material properties of the 3D compartmentalized tumoroids models with acellular, mesenchymal stromal cells or human lung fibroblasts containing stroma measured on day 21. (A) Average stiffness of 3D compartmentalized tumoroids (shear elastic modulus, G′), (B) Phase angle (δ) chart of 3D compartmentalized tumoroids. *n* = 4 for all samples, with significance determined through One‐way ANOVA and Tukey's multiple comparisons. Data is shown in graphs as the mean and standard deviation of the mean. **p‐value < 0.01.

The presence of HNLF in the dense collagen gels (21 days) causes a softening of the scaffold, to 636.03 ± 24.4 Pa compared to acellular gels at 749.2 ± 54.5. The MSCs have the most pronounced effect on softening the matrix, measuring 422.83 ± 99.22 Pa (Figure 3A). In spite of the observed reduction in stiffness, there was a slight increase in the phase angle across the cellular gels, indicating a tendency toward more liquid‐like behavior. The phase angle for acellular gels was found to be 10.73° ± 1.55°, compared to 11.64° ± 0.98° for gels containing human lung fibroblasts and 12.99° ± 0.86° for gels with mesenchymal stromal cells (Figure 3B). These observations suggest that while the inclusion of cells reduces the overall stiffness of the gel, it also induces microstructural changes within the gels and alters the viscoelastic balance of the matrix, which implies that the cellular gels possess a higher capacity for reversible deformation under strain compared to acellular gels.

### 3.3 Endothelial Cells and the Highly Metastatic Breast Cancer Cell Line, MDA‐MB‐231, Are More Susceptible to ILKI Treatment

**FIGURE 4:**
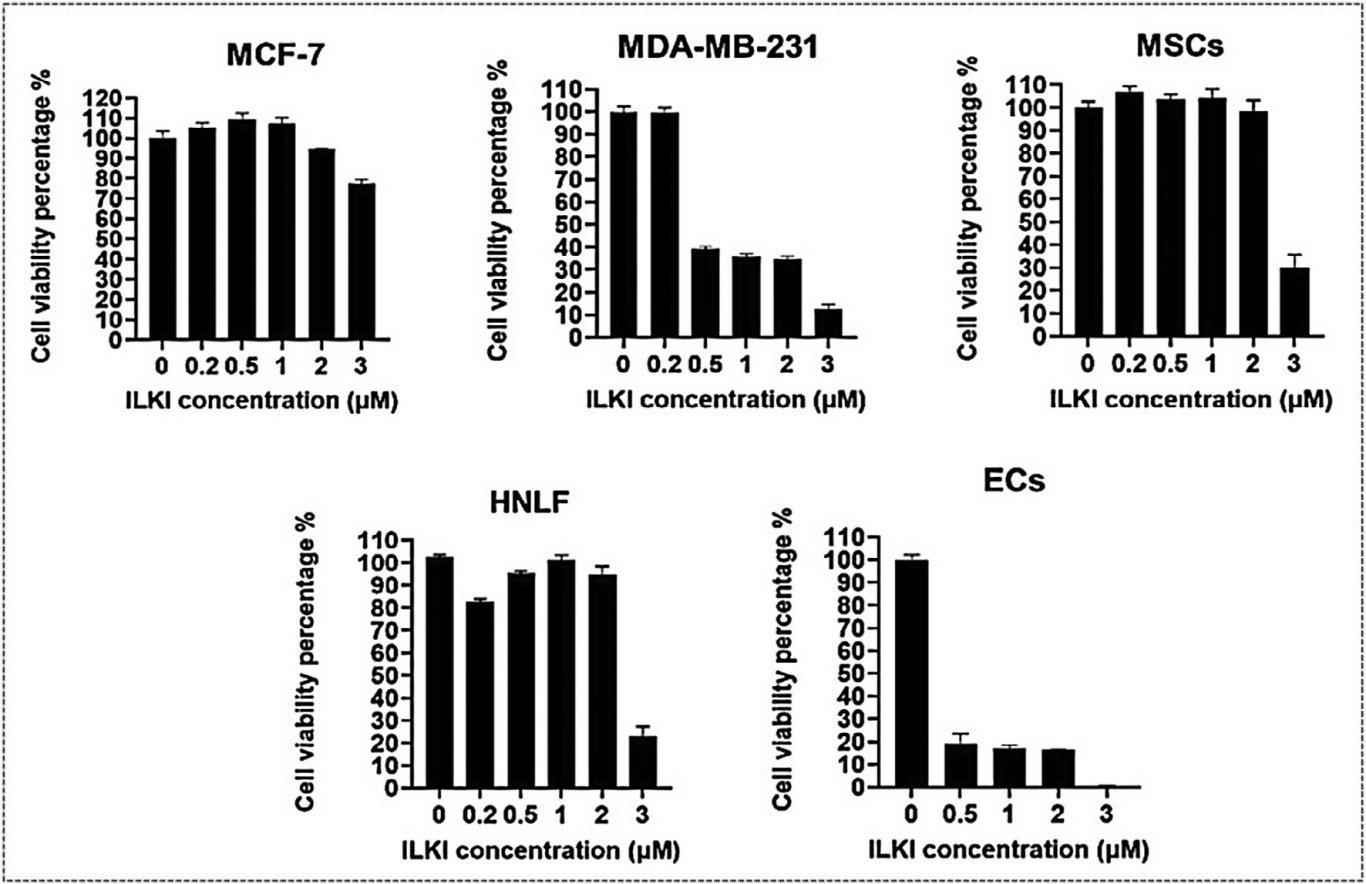
ILKI efficacy in 2D cultured breast cancer cells and healthy stromal cells using CellTiter‐Glo 3D Viability Assay, shown as graphs of cell viability percentage. Data are presented as the mean and standard error of the mean.

Treatment with ILKI at concentrations ranging from 0.2 to 3 μM showed that only the maximum concentration (3 μM) was effective for mesenchymal stem cells, human lung fibroblasts, and MCF‐7 cancer cells, yielding mean cell viability percentages of 29.8%, 22.9%, and 77.7%, respectively. In contrast, MDA‐MB‐231 cancer cells and endothelial cells were significantly more susceptible to the cytotoxic effects of increasing ILKI concentrations as shown in Figure 4.

### 3.4 Therapeutic Efficacy in 3D

#### 3.4.1 Breast Cancer Surrounded by a Primary Stromal Compartment: ILKI Targets MCF‐7 Cancer Cells More Than Stromal Cells, Compared to MDA‐MB‐231 Cells, Where Stromal Cells Are Targeted

**FIGURE 5:**
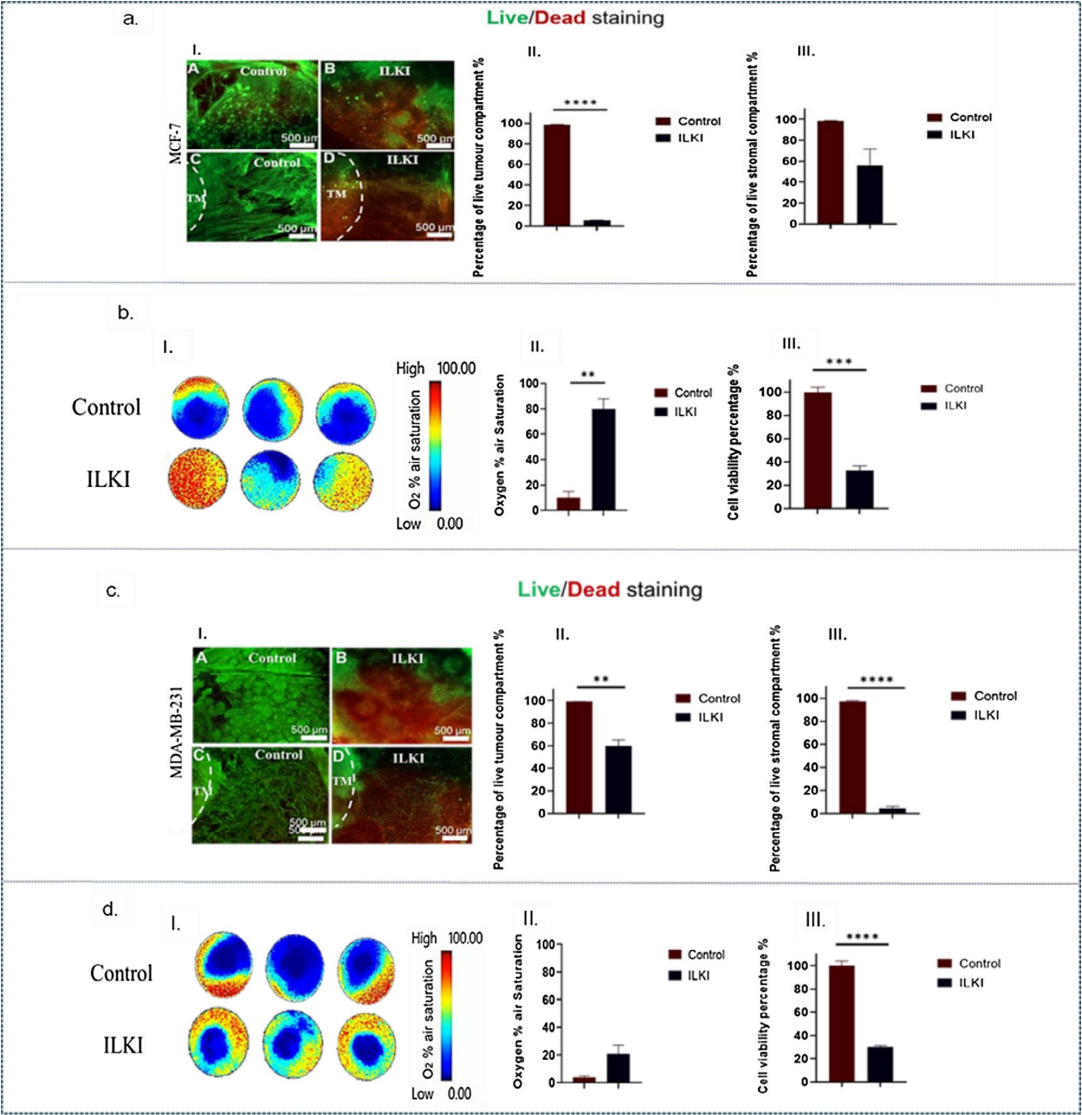
Efficacy of ILKI treatment in 3D compartmentalized tumoroids with a primary stromal compartment. (a.I) shows fluorescence images of MCF‐7 3D compartmentalized tumoroids stained with Live/Dead dyes, where A and C represent control group and B and D represent group treated with10 μM of ILKI. (a.II) shows the corresponding graph of the quantified percentage of live cells in the tumor compartment. (a.III) shows the corresponding graph of the quantified percentage of live cells in the stromal compartment. (b.I) represents pseudocolor images that depict the real‐time monitoring of oxygen gradient levels in MCF‐7 3D compartmentalized tumoroids. (b.II) represents a graph that shows the recorded percentage of oxygen air saturation, and (b.III) shows a graph that indicates the percentage of cell viability. (c.I) shows fluorescence images of MDA‐MB‐231 3D compartmentalized tumoroids stained with Live/Dead dyes, where A and C represent control group and B and D represent group treated with10 μM of ILKI. (c.II) shows the corresponding graph of the quantified percentage of live cells in the tumor compartment. (c.III) shows the corresponding graph of the quantified percentage of live cells in the stromal compartment. (d.I) represents pseudocolor images that depict the real‐time monitoring of oxygen gradient levels in MDA‐MB‐231 3D compartmentalized tumoroids (see Video S1). (d.II) represents a graph that shows the recorded percentage of oxygen air saturation, and (d.III) shows a graph that indicates the percentage of cell viability. The analysis is based on *n* = 3, with significance determined through unpaired *t*‐test. Data is shown in graphs as the mean and standard error of the mean. *p*‐values: **< 0.01, *** < 0.001, ****< 0.0001. TM, tumor mass.

Treatment of MCF‐7 3D compartmentalized tumoroids with 10 μM of ILKI for 48 h resulted in a significant (*p*‐value < 0.0001) decrease in the percentage of live cancer cells in the TM (2.8%) compared to the control (98.8%). On the other hand, cells in the stromal compartment were less affected (55.8%) than the TM as shown in Figure 5a. The cell titer glo assay showed a significant (*p*‐value < 0.0001) reduction in cell viability to 32.5% of treated compartmentalized tumoroids compared to the control, which correlated with a statistically significant (*p*‐value = 0.001) improvement in oxygen levels to 79.6% compared to 10.1% in the control group (Figure 5b).

In MDA‐MB‐231 3D compartmentalized tumoroids, a significant (*p*‐value = 0.001) reduction in the proportion of live cancer cells in the TM (59.6%) of the ILKI‐treated group compared to the control (99.1%) was noticed, whereas the stromal compartment showed a severe decline in the percentage of live cells (4.3%) compared to the control (97.3%) as shown in Figure 5c. The cell titer glo assay showed a significant (*p*‐value < 0.0001) loss in cell viability (30%) compared to the control (100%). This was associated with an increase in oxygenation to 20.8% in comparison to the control (3.6%) as shown in Figure 5d. Efficacy of drug killing therefore corresponds to a reduction in the hypoxic core in real time.

#### 3.4.2 Breast Cancer Surrounded by a Metastatic Lung Stromal Compartment

**FIGURE 6:**
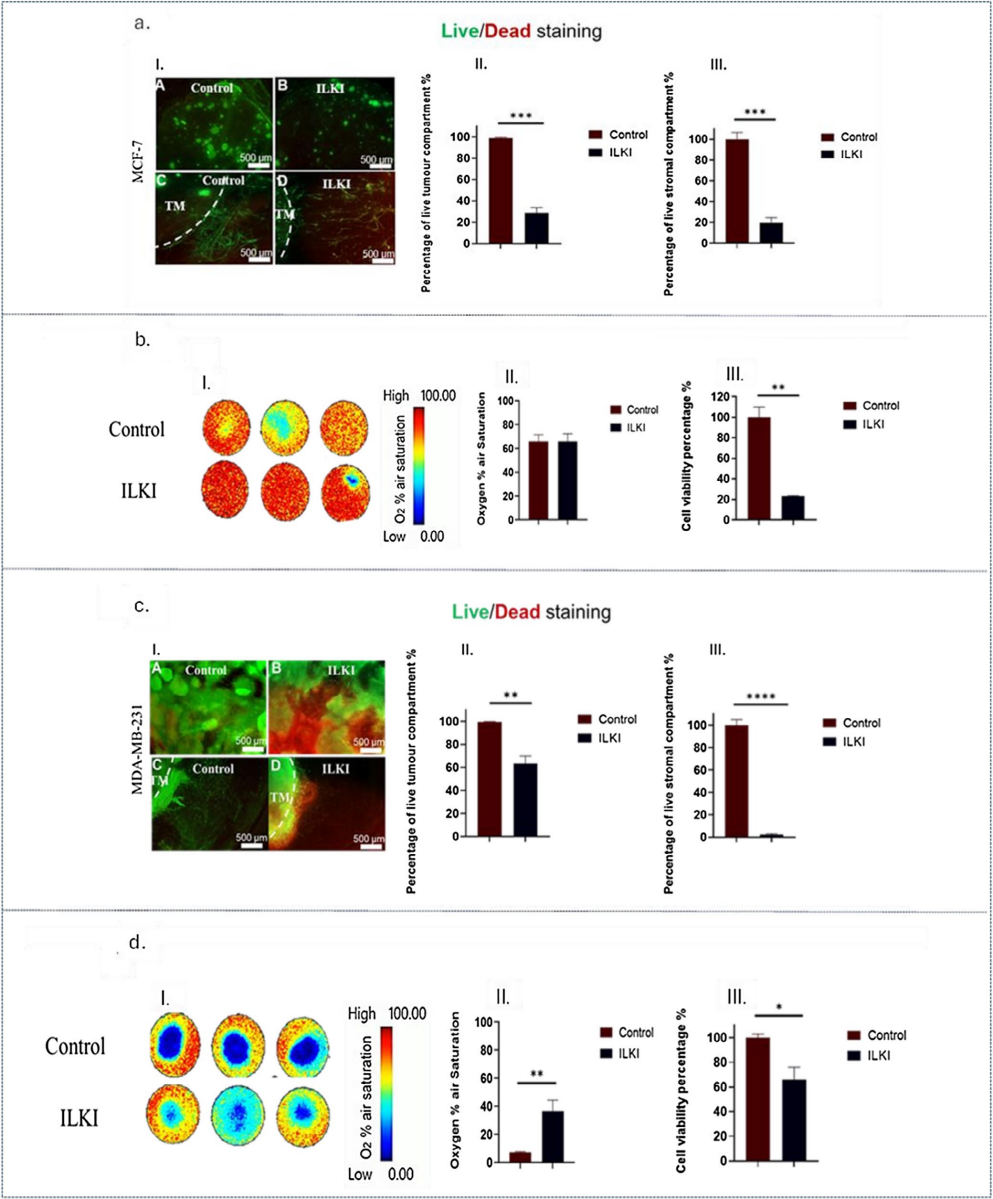
Efficacy of ILKI treatment in 3D compartmentalized tumoroids with a metastatic stromal compartment. (a.I) shows fluorescence images of MCF‐7 3D compartmentalized tumoroids stained with Live/Dead dyes, where A and C represent control group and B and D represent group treated with 10 μM of ILKI. (a.II) shows the corresponding graph of the quantified percentage of live cells in the tumor compartment. (a.III) shows the corresponding graph of the quantified percentage of live cells in the stromal compartment. (b.I) represents pseudocolor images that depict the real‐time monitoring of oxygen gradient levels in MCF‐7 3D compartmentalized tumoroids. (b.II) represents a graph that shows the recorded percentage of oxygen air saturation, and (b.III) shows a graph that indicates the percentage of cell viability. (c.I) shows fluorescence images of MDA‐MB‐231 3D compartmentalized tumoroids stained with Live/Dead dyes, where A and C represent control group and B and D represent group treated with 10 μM of ILKI. (c.II) shows the corresponding graph of the quantified percentage of live cells in the tumor compartment. (c.III) shows the corresponding graph of the quantified percentage of live cells in the stromal compartment. (d.I) represents pseudocolor images that depict the real‐time monitoring of oxygen gradient levels in MDA‐MB‐231 3D compartmentalized tumoroids (see Video S2). (d.II) represents a graph that shows the recorded percentage of oxygen air saturation, and (d.III) shows a graph that indicates the percentage of cell viability. The analysis is based on *n* = 3, with significance determined through unpaired *t*‐test. Data is shown in graphs as the mean and standard error of the mean. *p*‐values: *< 0.05, **< 0.01, ****< 0.0001. TM, tumor mass.

In MCF‐7 3D breast compartmentalized tumoroids, treatment with 10 μM of ILKI for 48 h caused a significant (*p*‐value = 0.0001) decline in the percentage of live cancer cells in the TM (28.7%) compared to the control (99.1%). In addition, there was a significant (*p*‐value = 0.0006) drop in the percentage of live cells in the stromal compartment to 19.7% compared to 100% in the untreated group as shown in Figure 6a. The overall cell viability in these tumoroids decreased to 22.9% in contrast to 100% in the control group. Nevertheless, real‐time oxygen monitoring showed a similar oxygen % air saturation in the control and treated groups. It is noteworthy that the baseline oxygen levels in the untreated control group remained quite stable, unlike those in the MCF‐7 3D compartmentalized tumoroids with the primary stromal compartment (Figure 6b).

In MDA‐MB‐231 3D compartmentalized tumoroids, findings of live‐dead imaging exhibited that treatment with 10 μM of ILKI for 48 h resulted in a significant (*p*‐value = 0.005) reduction in the proportion of live cancer cells in the TM to 63.1% compared to 99.4% in the control group. Only 2.2% of cells in the stromal compartment were still viable following this treatment (Figure 6c). Moreover, the results of the cell titer glo assay showed a 66% viability in the treated group compared to 99.9% in the control group. This correlated with a significant improvement in oxygen levels to 36.6% compared to 7.3% in the control group as shown in Figure 6d.

## 4 Discussion

Solid tumors such as breast cancer typically grow in a 3D configuration in vivo. 3D cell culture models promote cell–cell/cell‐ECM interactions, effectively simulate hypoxic conditions and provide a more accurate representation of drug response compared to 2D culture [18], offering biomimetic platforms for assessing therapeutic responses and reducing reliance on animal models [19]. Fabrication of 3D compartmentalized tumoroids enables replication of physiological matrix/tissue stiffness, which substantially contributes to providing the essential biophysical and biochemical cues for in vivo‐like cell behavior. This study investigated the therapeutic efficacy of an ILKI for breast cancer treatment utilizing an advanced 3D compartmentalized breast cancer model with either a primary or a metastatic stromal compartment, demonstrated by the findings of cell titer glo. Overall, ILKI treatment caused significant cytotoxicity in both MCF‐7 and MDA‐MB‐231 3D compartmentalized tumoroids.

3D tumoroids comprising MSCs facilitated vascular network formation compared to those comprising HNLF (Figure 2.1) which is consistent with other studies [20, 21, 22]. This can occur through multiple mechanisms such as secretion of many pro‐angiogenic factors (VEGF, Angiopoietin‐1/Tie2, FGF, PDGF, TGF‐β, SDF‐1, HGF, and IGF‐1) which significantly enhance the growth and viability of endothelial cells [23] and differentiation into ECs [21]. A study showed that MSCs expressed higher levels of VEGF and FGF4 compared to lung fibroblasts [24], which justifies the findings in this study (Figure 2.1). The tumor cell secretome is critical in determining angiogenic potential. For instance, MCF‐7 exosomes significantly upregulate CCL5, a chemokine known to promote angiogenesis, in comparison to MDA‐MB‐231 exosomes [25]. Additionally, exposure of HUVECs to MDA‐MB‐231 (claudin‐low TNBC subtype)‐derived conditioned medium causes a notable reduction in proliferation, significant morphological changes, stress fiber formation, and the disruption of inter‐endothelial junctions compared to HUVEC cells treated with the conditioned medium of MCF‐7 cells (luminal A BC subtype) [26, 27]. MDA‐MB‐231 cells generate greater contractile forces and degrade the ECM through MMPs 2, 7, and 8, potentially disrupting any established vascular patterns, while MCF‐7 cells preserve ECM integrity, enabling vessels to remain stable and interconnected [28]. Tumor spread relies on vascularization, which can occur through vasculogenesis (formation of new blood vessels from newly differentiated endothelial cells), angiogenesis (development of blood vessels from those that already exist), or vasculogenic mimicry (cancer cells can form structures that resemble blood vessels) [29]. However, the breast tumor phenotype will dictate which processes prevail. Studies have shown that MCF‐7 cancer cells generate more developed CD31‐positive vascular structures, consistent with vasculogenesis, whereas MDA‐MB‐231 cancer cells depend more on VM, producing poorly formed CD31‐negative vascular channels that are predominantly lined with tumor cells [30], which is in accordance with the findings herein.

Breast cancer invasion was significantly higher in 3D breast tumoroids with a metastatic stromal compartment comprising HNLF and ECs compared to those with a primary stromal compartment containing MSCs and ECs (Figure 2.2), despite the latter's superior promotion of vascular networks formation. Oxygen consumption was higher in 3D breast tumoroids with a primary stromal compartment in comparison to those with a metastatic stromal compartment (Figure 2.3). This highlights the fact that the TME extends beyond blood vessel density and that the metastatic process is highly complex. Fibroblasts and MSCs appear to play different roles within the TME, influencing the invasive behavior of tumor cells. Human lung fibroblasts, especially when activated into CAFs, secrete cytokines and growth factors (like TGF‐β, IL‐6, PDGF, EGF, HGF, CXCL‐12), ECM proteins, and remodeling enzymes like MMPs and pro‐invasive signals that potentiate cancer stemness and metastasis [31, 32, 33, 34, 35]. MSCs tend to support vascularization but have more variable, sometimes suppressive, effects on cancer invasion depending on the tumor subtype and ECM context. This is particularly evident where tumoroids with a primary stromal compartment reveal increased MCF‐7 TM growth and invasiveness in contrast to MDA‐MB‐231, which is supported by several studies [36, 37, 38, 39, 40, 41, 42].

ILKI treatment of 2D cultured breast cancer cells and stromal cells revealed that MDA‐MB‐231 cancer cells and ECs were particularly sensitive to lower ILKI concentrations, whereas MSCs and HNLF experienced a substantial decline in cell viability only at a concentration of 3 μM. MCF‐7 cancer cells were the least affected by the increasing concentrations of ILKI (Figure 4). A research study reported a higher ILK expression by MDA‐MB‐231 cells compared to MCF‐7 cells, which is consistent with the increased responsiveness of MDA‐MB‐231 to ILKI presented herein. On the other hand, no direct comparisons of ILK expression levels in MSCs, HNLF, and ECs were studied elsewhere. However, ILK supports EC survival under stress conditions like hypoxia via PI3K/Akt [43], maintains MSCs' survival and enhances their angiogenic potential [44], and drives fibroblast survival, migration, activation, and differentiation into myofibroblasts. TGF‐β is a major regulator of ILK expression and activity in lung fibroblasts, with TGF‐β signaling leading to an upregulation of ILK, thereby promoting fibroblast activation and ECM production [45].

ILKI efficacy in 3D breast tumor models with different TME components highlights the importance of dynamic cell‐ECM in driving integrin signaling, where physical cues from the ECM influence integrin activation and the signaling cascades involving ILK, leading to ILK expression and activity patterns that are more representative of the in vivo tumors. Studies have shown significantly altered integrin expression and downstream signaling pathways in 3D cultures [46, 47, 48] compared to 2D cultures, impacting proliferation, survival, and drug resistance of breast cancer cells. ILK functions as a critical molecular component in the dynamics of cell‐matrix interactions, enabling cell adhesion and fostering anchorage‐dependent growth [49]. Key downstream targets of ILK include Akt/PKB, GSK3β, β‐catenin, p44/42 MAP kinases (ERK1/2), the myosin light chain (MLC), and Merlin's phosphatase MYPT‐1, which is involved in the Hippo signaling pathway [50, 51]. ILK connects integrins to the actin cytoskeleton and is crucial in various cellular processes, including proliferation, differentiation, migration, invasion, and angiogenesis [49]. Herein, ILKI was significantly more effective in MDA‐MB‐231 3D compartmentalized tumoroids with a primary stromal compartment than those with a metastatic stromal compartment (Figures 5 and 6), while it was slightly more effective in 3D compartmentalized tumoroids of MCF‐7 with metastatic stromal compartment compared to those with a primary stromal compartment (Figures 5 and 6) as demonstrated by the findings of the CellTiter‐Glo. Moreover, live‐dead imaging showed that the stromal compartment was substantially more affected by ILKI in the MDA‐MB‐231 3D compartmentalized tumoroids compared to MCF‐7 3D compartmentalized tumoroids (Figures 5 and 6). HLNF‐induced matrix remodeling resulted in stiffer collagen scaffolds compared to MSC scaffold remodeling (Figure 3). ILK functions as a key mechanosensor, translating external mechanical cues (like ECM stiffness/density) into intracellular biochemical signals that regulate survival and invasion. Pang et al. reported that a stiff microenvironment promoted ILK expression and the development of breast cancer stem‐like cell populations among MDA‐MB‐231 cells [52]. Nevertheless, it is worth mentioning that this was tested as a monoculture 3D model of breast cancer. While ILK expression is largely determined by the composition and stiffness of ECM [53], ILK activity is also regulated by a complex interplay of biochemical and cellular cues within the TME such as growth factors and cytokines [45], hypoxia [52], inflammation [54], oncogenic signaling (oncogenes and tumor suppressor genes) [55] and direct protein–protein interactions (PINCH, parvin, and various adaptors and phosphatases) [56]. These factors often work in conjunction with mechanical signals to influence cancer cell behavior. Our study, to the best of our knowledge is the first to explore ECM remodeling by different stromal cells in 3D breast cancer model and how they could influence the response to an ILK inhibitor. Although the concept established by Pang et al. posits ILK as a central mediator of stiffness‐dependent oncogenesis, our physiologically relevant co‐culture model reveals that in a complex tumor microenvironment, the cellular origin of the stroma is a critical variable. The biochemical and architectural signature imparted by a specific stromal cell type can decouple the expected relationship between bulk stiffness and ILK dependency. Consequently, the therapeutic vulnerability to ILK inhibition is not universal but is profoundly influenced by the cellular context, specifically the breast cancer subtype and the originating stromal cell driving ECM remodeling.

We postulate that the differential therapeutic effect of ILKI in MDA‐MB‐231 and MCF‐7 3D tumoroid models with distinct stromal compositions can be explained by the interplay between stromal cell and cancer cell type [57], ECM remodeling, and ILK‐driven signaling pathways. We also hypothesize that the pro‐tumorigenic signaling pathways of MSCs and HNLFs are probably distinct and the dependence on ILK in stromal cells is thought to differ based on breast cancer subtype. The MDA‐MB‐231 cell line has an inherently aggressive phenotype driven by a self‐sustaining autocrine loop involving ILK, IL‐6, and NF‐κB [54, 58]. MSCs promote tumor growth largely through paracrine signaling, where they secrete factors that influence cancer cells [59]. As previously mentioned, their ILK activity supports their survival and enables them to produce these factors. Accordingly, MSCs' pro‐tumorigenic signaling is highly convergent with and dependent on the ILK signaling axis [60], creating a synergistic therapeutic vulnerability where the inhibitor targets a central, shared pathway in both the malignant and stromal cell populations. Therefore, the ILK inhibitor is highly effective as it not only blocks matrix stiffness‐induced pathways but also MSCs‐driven ILK‐dependent pathways. In contrast to MSCs, fibroblasts engage in both paracrine signaling and direct cell–cell contact, matrix‐mediated physical interactions with cancer cells [61, 62]. In the HNLF‐containing model, the HNLFs generate a stiffer, more mechanically driven microenvironment (Figure 6) that activates ILK and other possible ILK‐independent driven mechanisms involved in the overall pro‐tumorigenic signaling such as small GTPase RhoA and its effector ROCK, which regulate the actin cytoskeleton and contractility. This pathway, which can operate downstream of or in parallel to ILK, directly mediates the nuclear translocation of key transcription factors like YAP/TAZ and MRTF [63, 64]. Fibroblasts can sense mechanical cues via ILK‐integrin complexes and a variety of other mechanosensors and signaling molecules, including focal adhesion kinase (FAK), Src, Talin–Kindlin and components of the Hippo pathway [65, 66]. In a rigid environment, these pathways may become constitutively active in addition to the dominance of a parallel, non‐ILK pathway (COX‐2/PGE2 axis), making the cell less dependent on ILK activity [67]. Hence, the ILK inhibitor would still exert effects but less pronounced than in MSC‐rich models, where ILK sustains a more complex, biochemically driven pro‐tumorigenic network.

Unlike aggressive MDA‐MB‐231 cells, MCF‐7 cells lack pro‐tumorigenic loops and show low IL‐6 and COX‐2/PGE2 basal expression [54, 58]. Because of this different baseline, the role of stromal cells is not to amplify an existing aggressive program, but mostly to actively reprogram the MCF‐7 cell's phenotype into a new, more aggressive one. MCF‐7 cells are less reliant on ILK and mechano‐transduction for their primary oncogenic drives (proliferation and survival), which are mainly dictated by ER signaling [68]. Co‐culture with HNLFs remodels the ECM, driving integrin clustering and ILK activation via FAK–PI3K–AKT [69]. ILK then converts these mechanical cues into biochemical signals that promote cancer cell proliferation [70], survival, EMT‐like transition toward a more invasive phenotype, increasing MCF‐7 cells' reliance on ILK, and making its inhibition more disruptive in this dynamic, physically demanding environment. Thus, the enhanced therapeutic effect of ILK inhibitors in MCF‐7 compartmentalized tumoroids containing HNLFs compared to MSCs could be due to the differential signaling dependencies of MCF‐7 cells. The softer microenvironment generated by MSCs in the MCF‐7 3D compartmentalized tumoroids does not provide the same mechanical cues for EMT induction. Since MCF‐7 cells are less migratory and the ECM is less stiff, their pro‐invasive and survival signals are less reliant on ILK‐mediated mechano‐transduction. Therefore, the effect of an ILK inhibitor is less prominent because it is not targeting a key, stiffness‐induced dependency of cancer cells. Furthermore, Dittmer et al. showed that MSCs sensitize both MCF‐7 and MDA‐MB‐231 to kinase inhibitors, but the effect was especially pronounced in MDA‐MB‐231 due to their aggressive mesenchymal phenotype and reliance on integrin/ILK signaling for migration and EMT‐like behavior [71].

ILK pathways are linked to multidrug resistance in breast cancer [72, 73], with both in vitro [58, 70] and in vivo [74, 75] studies showing elevated ILK expression associated with disease progression. Patients' tissue samples revealed that ILK mRNA expression was significantly elevated in breast cancer tissues compared to the adjacent normal tissues [76]. Some preclinical studies demonstrate that ILKI suppress breast tumor growth and proliferation whether as a single agent [77, 78] or in combination with other drugs [73, 79, 80]. Furthermore, ILKI induces significantly more apoptosis in breast cancer cells compared to normal breast epithelial cells, fibroblasts, or vascular smooth muscle cells [81], denoting selective targeting of breast cancer sparing normal tissues which would reduce off‐target side effects and potentially enhance patients' compliance. To sum up, the varying response to ILK inhibition based on breast cancer subtype and stromal cell composition underscores the complexity of cancer and the need for precision medicine. These findings pave the way for a new generation of targeted and combination therapies that consider the TME's heterogeneity to improve treatment outcomes and overcome therapeutic resistance. Additionally, diagnostic tools could be developed to profile the stromal cell populations and their ILK pathway activity within a patient's tumor. This would enable personalized treatment strategies, ensuring the right therapy for the right patient.

### 4.1 Limitation of the Study

The ILKI used herein OSU‐T315 is reported to show high degree of specificity for ILK at 5 μM in a study that involved conducting a kinase profiling assay against a panel of 20 recombinant kinases [82]. Additionally, the same study demonstrated that OSU‐T315 modulated the phosphorylation of signaling proteins AKT (Ser473), GSK‐3β, and myosin light chain similarly to shRNA‐mediated knockdown of ILK which proves that OSU‐T315 effectively inhibits ILK activity [82]. OSU‐T315 may cause cell death through apoptosis and autophagy through ILK inhibition at 0–4 μM for 24 h. It also exhibits dose‐dependent suppressive effects on the levels of phospho‐ERK1/2 and phospho‐p38 and can reduce YB‐1, HER2, and EGFR expression [82]. Because the profiling concentration (5 μM) is below the concentration used in the present study (10 μM), and because small‐molecule inhibitors can exhibit concentration‐dependent off‐target activity, we note that some off‐target contributions cannot be excluded at the higher concentration used here. These potential for off‐target effects from the OSU‐T315 cannot be fully excluded without genetic validation. Future studies employing ILK genetic knockout models, including control cells and silenced cells with and without the ILKI, will be necessary to definitively confirm these findings.

## 5 Conclusion and Future Directions

The TME is a key contributor to breast cancer progression. Stromal components such as fibroblasts, mesenchymal stromal cells, immune cells, and the ECM provide biochemical cues (e.g., cytokines, growth factors) and mechanical signals (e.g., matrix stiffness) that drive malignancy. ILK is a signaling hub that integrates these external inputs into pro‐tumorigenic responses. Because ILK links tumor cell‐intrinsic programs with microenvironmental support, it represents both a marker of TME influence and a potential therapeutic target in breast cancer. This study employed a sophisticated 3D breast tumor‐stroma model to investigate the therapeutic effectiveness of an ILKI. A differential response among different breast cancer subtypes to ILKI treatment was demonstrated. Utilizing a model with a central TM and a surrounding stromal compartment, we were able to identify varying responses to treatment between the TM and the stromal components. The study also examined the effect of different 3D stroma tissues on treatment efficacy, which, to the best of our knowledge, has not been previously investigated. For MDA‐MB‐231 3D compartmentalized tumoroids, the applied treatment was substantially more effective in the tumoroids comprising a primary breast stromal compartment compared to those with a metastatic lung stroma compartment, whereas in MCF‐7 3D compartmentalized tumoroids, treatment efficacy in models comprising a metastatic lung stroma compartment was observed. These findings underscore the potential of TME‐targeted therapies in breast cancer. Further research could entail the evaluation of ILKI's effectiveness within 3D models that utilize patient‐derived tissues, comprising both cancer cells and stromal cells, such as cancer‐associated fibroblasts, tumor‐associated macrophages, and tumor‐associated endothelial cells from both primary and metastatic sites. Additionally, more studies are essential to explore the underlying mechanisms of ILKI's efficacy in these 3D models through conducting spatial analysis such as transcriptomics, proteomics and epigenomics. Furthermore, a combination of ILKI with other ECM targeting agents like Collagen/Hyaluronic acid synthesis inhibitors and Hyaluronic acid degradation, angiogenesis or hypoxia targeting agents could be tested.

## Author Contributions

S.T.R. executed experiments, performed data analysis, drafted the original manuscript, and constructed the figures. A.U. carried out rheological assessments and aided in writing the manuscript. U.C. and A.J.M. were involved in conceptualizing the study and editing the manuscript.

## Funding

This work was supported by the Egyptian Ministry of Higher Education & Scientific Research and the EPSRC (EP/T026324/1).

## Ethics Statement

The authors have nothing to report.

## Consent

The authors have nothing to report.

## Conflicts of Interest

The authors declare no conflicts of interest.

## Supporting information


**Video S1:** Real‐time oxygen gradient monitoring in bioengineered MDA‐MB‐231 3D compartmentalized tumoroids with a primary stromal compartment over 24 h period. Hypoxic tumor is shown in the control untreated group represented by deep blue color. Integrin‐linked kinase inhibitor treatment improved oxygen saturation from 3.6% to 20.8%.

**Video S2:** Real‐time oxygen gradient monitoring in bioengineered MDA‐MB‐231 3D compartmentalized tumoroids with a metastatic stromal compartment over 24 h period. Alleviation of hypoxia is noted in the group treated with Integrin‐linked kinase inhibitor from 7.3% oxygen saturation to 36.6% oxygen saturation, represented by the light blue color.

## Data Availability

The datasets used and/or analyzed during the current study are available from the corresponding author on reasonable request.
